# Extracellular vesicle therapeutics in Alzheimer’s disease: mechanisms, progress, and prospects

**DOI:** 10.20517/evcna.2025.158

**Published:** 2026-03-30

**Authors:** Yiduo Fang, Xiaotong Hao, Bo Fang

**Affiliations:** ^1^Department of Anesthesiology, The First Hospital of China Medical University, Shenyang 110001, Liaoning, China.; ^2^First Clinical Medical College, Shenyang Medical College, Shenyang110034, Liaoning, China.; ^#^These authors contributed equally to this work.

**Keywords:** Alzheimer’s disease, extracellular vesicles, mesenchymal stem cells, proteomics, traditional Chinese medicine

## Abstract

Alzheimer’s disease (AD) is the most prevalent neurodegenerative disorder worldwide, characterized by progressive cognitive decline and a current lack of effective curative treatments. In recent years, extracellular vesicle (EV) therapies have emerged as a cutting-edge approach in AD research due to their unique biological properties. This review systematically examines the role of EVs in the pathogenesis of AD, including their cellular origins and functional attributes. We detail the molecular mechanisms mediated by EVs that influence AD progression and summarize the latest advancements in EV-based therapeutic strategies. Additionally, the review addresses the challenges faced in translating these therapies from bench to bedside, such as standardization, delivery optimization, and safety concerns. Future research directions are discussed to foster the development of innovative and effective treatments for AD. By providing a comprehensive overview, this article aims to lay a theoretical foundation and offer valuable insights for the advancement of novel therapeutic interventions targeting AD.

## INTRODUCTION

Alzheimer’s disease (AD) stands as the most prevalent neurodegenerative disorder globally, primarily characterized by progressive cognitive decline, memory impairment, and behavioral abnormalities that severely compromise patients’ quality of life. Epidemiological data estimate that over 55 million people worldwide are living with dementia, with AD accounting for approximately 60%-70% of these cases^[1-2]^. The pathological hallmarks of AD include extracellular amyloid-beta (Aβ) plaques and intracellular neurofibrillary tangles (NFTs) composed of hyperphosphorylated tau protein, which accumulate in the brain over decades before clinical symptoms manifest^[3-4]^. The complex multifactorial nature of AD involves not only protein aggregation but also synaptic dysfunction^[5]^, necroptosis^[6]^, mitochondrial impairment^[7]^, neuroinflammation^[8]^ and disrupted proteostasis^[9]^, all contributing to neuronal loss and network disintegration. Therefore, despite extensive research, effective treatments remain elusive. The current drug therapies mainly provide symptom relief but are unable to halt or reverse the progression of the disease^[10-11]^.

In recent years, the role of extracellular vesicles (EVs) has gained considerable attention in the context of AD pathogenesis and therapy. EVs are nanoscale lipid bilayer-enclosed vesicles secreted by virtually all cell types, including neurons, glial cells, and stem cells, and serve as critical mediators of intercellular communication by transferring proteins, lipids, and nucleic acids. Their unique biological properties, such as intrinsic biocompatibility, ability to cross the blood-brain barrier (BBB), and low immunogenicity, render them promising candidates as natural drug delivery vehicles and diagnostic biomarkers^[12-13]^.

In AD, EVs exhibit dual characteristics as both pathological promoters and therapeutic agents. They contribute to disease progression by facilitating the intercellular transmission of pathogenic proteins such as Aβ and tau, thereby accelerating pathological spread. Conversely, EVs also play regulatory roles in neuroinflammation^[14-16]^, maintain neuronal homeostasis, and support synaptic plasticity^[14,17-18]^. Specific molecular cargoes carried by EVs can serve as biomarkers for early diagnosis and disease monitoring^[19-20]^. Furthermore, through genetic engineering, EVs can be engineered as precision delivery systems for loading therapeutic agents such as small-molecule drugs and nucleic acids, thereby opening new avenues for targeted therapy in AD^[21-22]^.

Despite these promising advances, several challenges remain in translating EV-based therapies to the clinic, including scalable production, standardization of isolation and characterization methods, efficient drug loading, and controlled targeting. Furthermore, while optimizing therapeutic strategies, it is crucial to aim for the maximal reduction of EV-mediated propagation of pathological proteins, thereby preventing the adverse sequelae associated with the progression of AD^[23]^. Therefore, it is crucial to understand the complex interactions between EV biosynthesis, cargo selection, and their functional roles in the pathology of AD.

This review aims to systematically elucidate the current application status of EV-based therapies in AD, with a focus on their mechanisms of action, proteomic profiles, preclinical and early clinical advances, as well as engineering and pretreatment strategies for EVs. Through in-depth analysis, it seeks to establish a comprehensive theoretical framework and research foundation for developing novel interventions against this devastating disease.

## BIOLOGICAL CHARACTERISTICS OF EVs AND THEIR ADVANTAGES IN AD

### Structure and composition of EVs

The diameter of EVs typically ranges between 30 and 150 nanometers. Their biogenesis begins with the inward budding of the plasma membrane to form early endosomes. These endosomes then mature into multivesicular bodies, which ultimately fuse with the plasma membrane, releasing the contained intraluminal vesicles as EVs^[24-25]^. Structurally, EVs exhibit a phospholipid bilayer membrane rich in cholesterol, sphingomyelin, glycosphingolipids, and phosphatidylserine, which confer membrane stability and influence their biophysical properties such as fluidity and membrane polarity^[26-27]^. Characteristic protein markers commonly enriched on EV membranes include tetraspanins (CD9, CD63, CD81), heat shock proteins, and membrane transport and fusion proteins, which are instrumental in EV identification and function^[28-29]^. Importantly, beyond these universal markers, EVs also carry distinctive molecules that reflect their cellular origin, enabling the identification and sorting of specific EV subpopulations. For instance, neural cell-derived EVs express cell-type-specific proteins, such as sodium/potassium-transporting ATPase subunit alpha-3 (ATP1A3) and neural cell adhesion molecule 1 (NCAM1) in excitatory neurons. They also express low-density lipoprotein receptor-related protein 1 (LRP1) and integrin alpha-6 (ITGA6) in astrocytes, and integrin alpha-M (ITGAM) and lymphocyte cytosolic protein 1 (LCP1) in microglia. They also express lysosomal-associated membrane protein 2 (LAMP2) and ferritin heavy chain 1 (FTH1) for oligodendrocytes^[30]^. These specific markers provide key molecular targets for immunocapture-based isolation of EVs from particular cell types. Among emerging platforms, Leprechaun technology represents a high-sensitivity approach that captures and analyzes individual EV particles directly from complex biological samples by immobilizing antibodies against specific markers on a chip. This method effectively avoids interference from common contaminants such as lipoproteins and protein aggregates, thereby enabling specific quantification of target EVs. Moreover, it allows parallel analysis of multiple cell-specific markers in a single experiment. Thus, Leprechaun technology provides a powerful tool for efficiently sorting EVs derived from distinct neural sources, such as neurons, astrocytes, and microglia^[31]^. Similarly, the internal cargo of EVs is highly heterogeneous and includes diverse bioactive molecules such as proteins, lipids, nucleic acids (including messenger RNAs (mRNAs), microRNAs (miRNAs), and other non-coding RNAs), and metabolites^[32]^. This composition is not static but is dynamically shaped by the cell of origin and the physiological or pathological context. Therefore, the biological function of EVs is determined by their composition, which also renders them valuable as diagnostic biomarkers^[33-35]^.

In summary, the sophisticated structure and composition of EVs underpin their multifaceted roles in intercellular communication and disease pathogenesis. Those released in a healthy context may support neuronal homeostasis, whereas vesicles from stressed or diseased cells often propagate pathology^[17,33]^. Understanding this bidirectional nature is essential for developing EV-based strategies that selectively inhibit their deleterious functions while harnessing their protective potential. Similarly, the inherent properties of EVs also make them exceptionally attractive as platforms for EV therapeutic strategies and as tools for diagnosis in neurodegenerative disorders such as AD.

### BBB crossing ability

**Figure 1:**
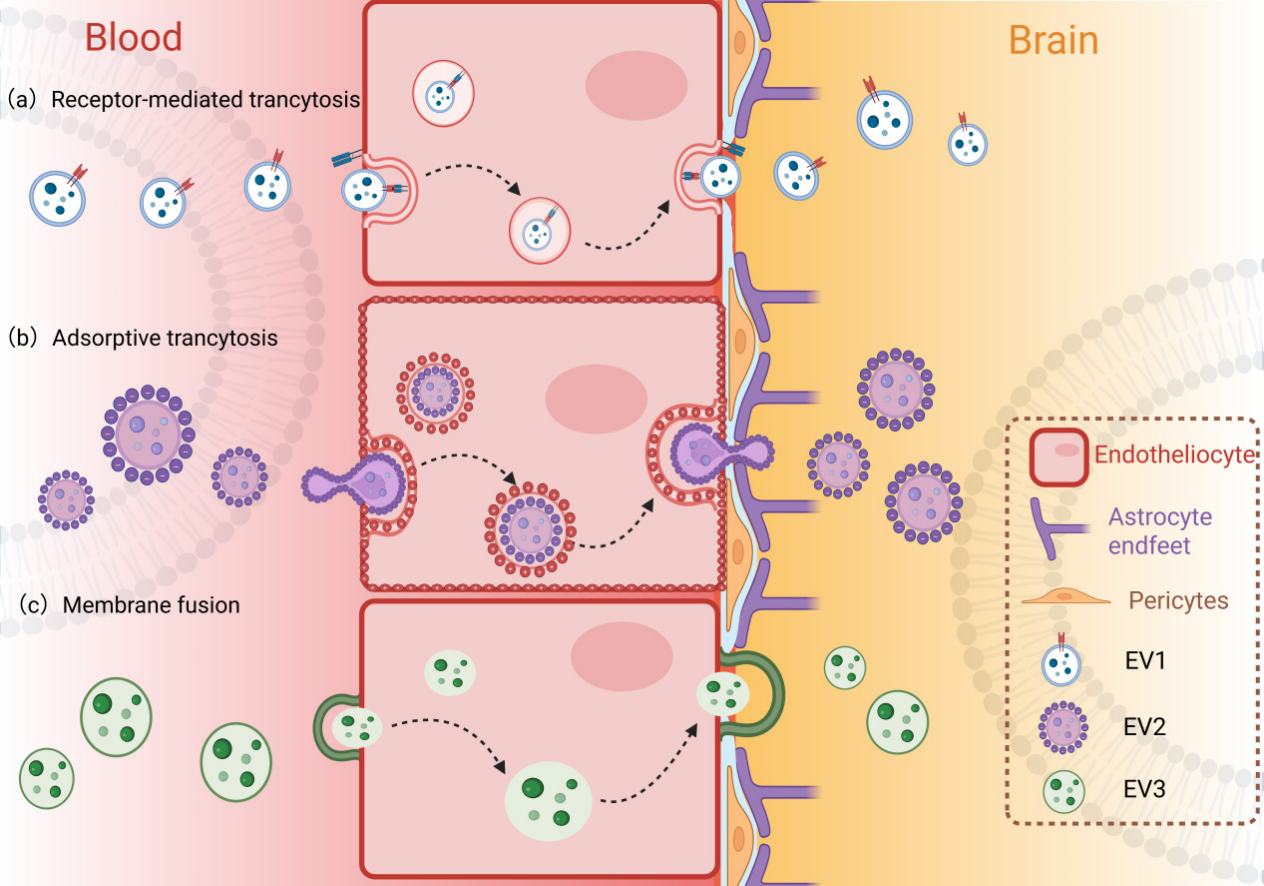
Three pathways for exosomal trafficking across the BBB into the brain. EV: Extracellular vesicle; BBB: blood-brain barrier. Created in BioRender. yinghan, h. (2025) https://BioRender.com/vfl6g0j

BBB is a highly selective barrier composed of brain microvascular endothelial cells, astrocytic end-feet, pericytes, and the extracellular matrix. It maintains central nervous system (CNS) homeostasis by tightly regulating the transport of molecules between the blood and the brain parenchyma^[36]^. Paradoxically, this intricate mechanism poses the foremost challenge for drug delivery to the brain, which severely restricts the therapeutic arsenal for a range of CNS diseases, such as AD. In this context, EVs have emerged as promising natural nanocarriers with an intrinsic ability to cross the BBB, offering a potential solution to this long-standing delivery challenge^[37]^. EVs, especially brain-derived EVs, can cross the BBB and enter the peripheral blood. The specific molecular information they carry provides a very promising source of biomarkers for detecting and monitoring CNS diseases from peripheral blood, which is of great value for the research of neurodegenerative diseases^[38]^ [Figure 1].

The ability of EVs to cross the BBB is mediated by several mechanisms, notably receptor-mediated transcytosis, adsorptive transcytosis, and membrane fusion^[39-41]^. EVs derived from different cell types across various species have been shown to cross the BBB both *in vitro* and *in vivo*, delivering cargo such as miRNAs, proteins, and drugs to target cells in the brain^[37,42]^. The transport rates and mechanisms can vary depending on the EV source, surface protein composition, and physiological or pathological conditions influencing BBB permeability^[43-46]^. Furthermore, EVs derived from pathological conditions, such as those from periodontitis or preeclampsia, have been found to alter BBB properties and permeability, suggesting that EVs can also modulate BBB function in disease states^[47-48]^.

Despite these advancements, there are still challenges in fully elucidating the precise mechanism by which EVs cross the BBB and in optimizing the engineering of EVs. Future research should focus on the molecular interactions between EVs and the components of the BBB, as well as the impact of pathological conditions on EV transport, in order to promote the development of EV modification techniques.

### Low immunogenicity and biosafety

EVs derived from autologous or allogeneic cells exhibit inherently low immunogenicity and excellent biocompatibility, rendering them highly suitable for therapeutic applications in AD. Their favorable safety profile stems primarily from their nanoscale vesicular structure, which lacks major histocompatibility complex (MHC) molecules and does not express surface antigens that typically trigger immune rejection, thereby minimizing the risk of adverse immune responses upon administration^[49-50]^. Mesenchymal stem cell (MSC)-derived EVs, in particular, have been extensively studied for their immunomodulatory properties and safety profile. They demonstrate no cytotoxic or immunogenic effects in various *in vitro* and *in vivo* models^[51-52]^. Repeated administration via nasal or intravenous routes remains safe, which is critical for managing chronic diseases such as AD^[53]^. Moreover, the lipid bilayer structure of EVs confers stability in circulation and protects their cargo from enzymatic degradation, further enhancing their biocompatibility and therapeutic efficacy^[54]^. 

## DIFFERENCES IN EVs ORIGINS AND FUNCTIONS

### MSC-EVs

MSC-EVs retain the beneficial properties of their parent cells while circumventing risks associated with cell-based therapies, including immunogenicity, tumorigenicity, and storage limitations^[55]^.

In the context of AD, MSC-EVs have demonstrated neuroprotective effects by reducing neuronal apoptosis, modulating neuroinflammation and autophagy. MSC-EVs can mitigate the accumulation of Aβ by conveying miRNAs such as miR-29c-3p, which targets beta-site amyloid precursor protein cleaving enzyme 1 (BACE1), and by modulating key signaling pathways such as Wingless-type MMTV integration site family (Wnt)/β-catenin. These actions ultimately reduce neuronal cell death and neuroinflammation^[56]^. Furthermore, MSC-EVs inhibit microglial activation, shifting their phenotype towards an anti-inflammatory state and reducing the release of pro-inflammatory factors such as IL-6 and TNF-α, thereby significantly ameliorating the AD-specific neuroinflammatory microenvironment^[53,57]^. Additionally, MSC-EVs promote autophagy via the phosphoinositide 3-kinase (PI3K)/protein kinase B (AKT)/mammalian target of rapamycin (mTOR) pathway, facilitating the clearance of hyperphosphorylated tau and Aβ, which in turn improves memory and alleviates neurological damage^[58]^.

MSCs can be isolated from a wide range of tissues, including the umbilical cord (UC), placenta (PL), adipose tissue (AT), and bone marrow (BM). The heterogeneity among MSCs from different tissue sources results in variations in the EVs they secrete. Proteomic analysis reveals that BM-MSC EV proteins are primarily involved in granulocyte activation and the regulation of cell migration. In contrast, AT-MSC and UC-MSC EV proteins are enriched in processes related to leukocyte activation and immune responses. Furthermore, UC-MSC EV proteins are also enriched in collagen metabolic processes. In terms of molecular function, both AT-MSC and UC-MSC EV proteins are significantly enriched in cell adhesion molecule binding, whereas BM-MSC EV proteins mainly participate in protein complex binding and integrin binding^[59]^. Compared with adult-derived MSCs, fetal-derived MSCs secrete EVs containing more proteins and possess stronger functional characteristics, including migration, cell development, and metabolic processes^[60]^.

### Neuronal and glial cell-derived EVs

Neuronal and glial cell-derived EVs are pivotal mediators in intercellular communication within the CNS, profoundly influencing neuronal signaling, synaptic plasticity, and inflammatory responses. These EVs reflect the functional states of their parent cells and participate in both physiological processes and disease mechanisms. Crucially, the pioneering work of isolating these cell-specific EVs from peripheral blood has established them as potent biomarkers for the preclinical stage of AD.

Neuronal EVs carry a unique protein repertoire that reflects intracellular core processes, and their functional properties are poised to modulate fundamental biological and pathological processes. The foundational study by Fiandaca *et al.* demonstrated that neuron-derived EVs from blood plasma contained elevated levels of pathogenic proteins such as Aβ 42 and p-T181-tau in individuals who would develop AD, detectable 1-10 years before clinical diagnosis, highlighting their early diagnostic potential^[61]^. As a new type of signaling system, neuronal EVs facilitate communication between neurons in a manner that is distinct from classical neurotransmitters^[62]^. These proteins regulate the spontaneous activity of neuronal networks by altering synchronous burst patterns and neuronal spontaneous discharge, thereby influencing synaptic plasticity and neuronal excitability^[63]^. Their release is dynamically regulated under various conditions. For example, chronic stress alters neuronal EV levels in both brain regions and plasma, with implications for stress-related pathophysiology^[64]^. Functionally, neuronal EVs contribute to Aβ clearance through ceramide-dependent release mechanisms, suggesting a neuroprotective role in AD^[65]^. Furthermore, neuron-derived EVs can drive conformational changes in Aβ to form non-toxic amyloid fibrils and promote its uptake by microglia^[15]^. However, some researchers have found that neuron-derived EVs isolated from the plasma of AD patients can impair neuronal viability in rats, indicating a potential neurotoxic effect^[66]^. Beyond modulating neuronal viability, EVs serve as potent vehicles for the intercellular transmission of proteopathic seeds. A pivotal study provides direct experimental evidence for this concept: When EVs isolated from the brains of AD patients were injected into the hippocampus of wild-type mice, human tau pathology was detected not only in ipsilateral hippocampus and thalamus but also prominently in contralateral hippocampus and thalamus two months post-injection. This result unequivocally demonstrates that AD-EVs can mediate the effective dissemination and seeding of pathological tau across hemispheres, along neural circuits, and even across species. Furthermore, hippocampal neurons in mice injected with these human AD-EVs exhibited significant dendritic beadings, a hallmark of neuronal stress and impending apoptosis, confirming the direct neurotoxic potential of the EV-borne tau seeds in driving neurodegeneration. Thus, EVs act not only as an “accelerator” for the intracerebral spread of AD pathology but also, by virtue of their ability to carry and protect intact proteopathic seeds, establish a potential basis for the inter-individual or cross-species transmission of the disease^[67]^. The EVs secreted by glial cells also make a significant contribution to the homeostasis and neuroinflammation of the CNS. Goetzl *et al.* established a method to immunocapture astrocyte-derived EVs (via glutamate aspartate transporter (GLAST)/excitatory amino acid transporter 1 (EAAT1)) from plasma, revealing significant alterations in complement proteins in AD, thereby identifying another critical cellular source of AD biomarkers^[68]^. Astrocyte-derived EVs have been shown to regulate synaptic plasticity and modulate inflammatory responses by transferring proteins, miRNAs, and lipids that influence neuronal and glial function^[69]^. When astrocytes phagocytose substantial amounts of Aβ, they subsequently release Aβ1-42 fibrils via EVs, which can induce apoptosis in surrounding neurons^[70]^. The role of microglial cell EVs is bidirectional. When in the M1 state, their EVs will downregulate the expression of ferroptosis suppressor proteins, thereby inducing ferroptosis in neurons and exacerbating neural damage. Conversely, in the M2 polarized state, microglial EVs deliver miR-124, which reduces neuronal apoptosis and alleviates brain injury^[71-72]^. Oligodendrocyte-derived EVs promote axonal integrity and support myelination by delivering trophic factors and molecular cargo, thereby providing crucial support for neuronal maintenance and regeneration^[73-74]^.

Overall, neuronal and glial EVs form an intricate communication network that regulates synaptic plasticity, neuronal survival, and inflammatory responses. They have both “protective” and “destruction” properties, offering promising biomarkers and therapeutic targets for neurodegenerative diseases.

### Peripheral immune cell-derived EVs

EVs derived from peripheral immune cells have emerged as key mediators linking systemic immunity to neuroinflammation in AD^[75]^. These EVs transport proteins, lipids, miRNAs, and even mitochondrial components across the BBB, allowing them to directly influence the CNS microenvironment. For instance, macrophage-derived EVs can exacerbate cognitive impairment in mice by promoting inflammation in both the peripheral blood and the CNS^[76]^. In contrast, EVs derived from non-activated macrophages can cross the BBB and deliver brain-derived neurotrophic factor (BDNF) to the brain, activating the receptor tropomyosin receptor kinase B (TrkB) and inhibiting cell apoptosis. This process is enhanced in the presence of brain inflammation^[77]^.

**Figure 2:**
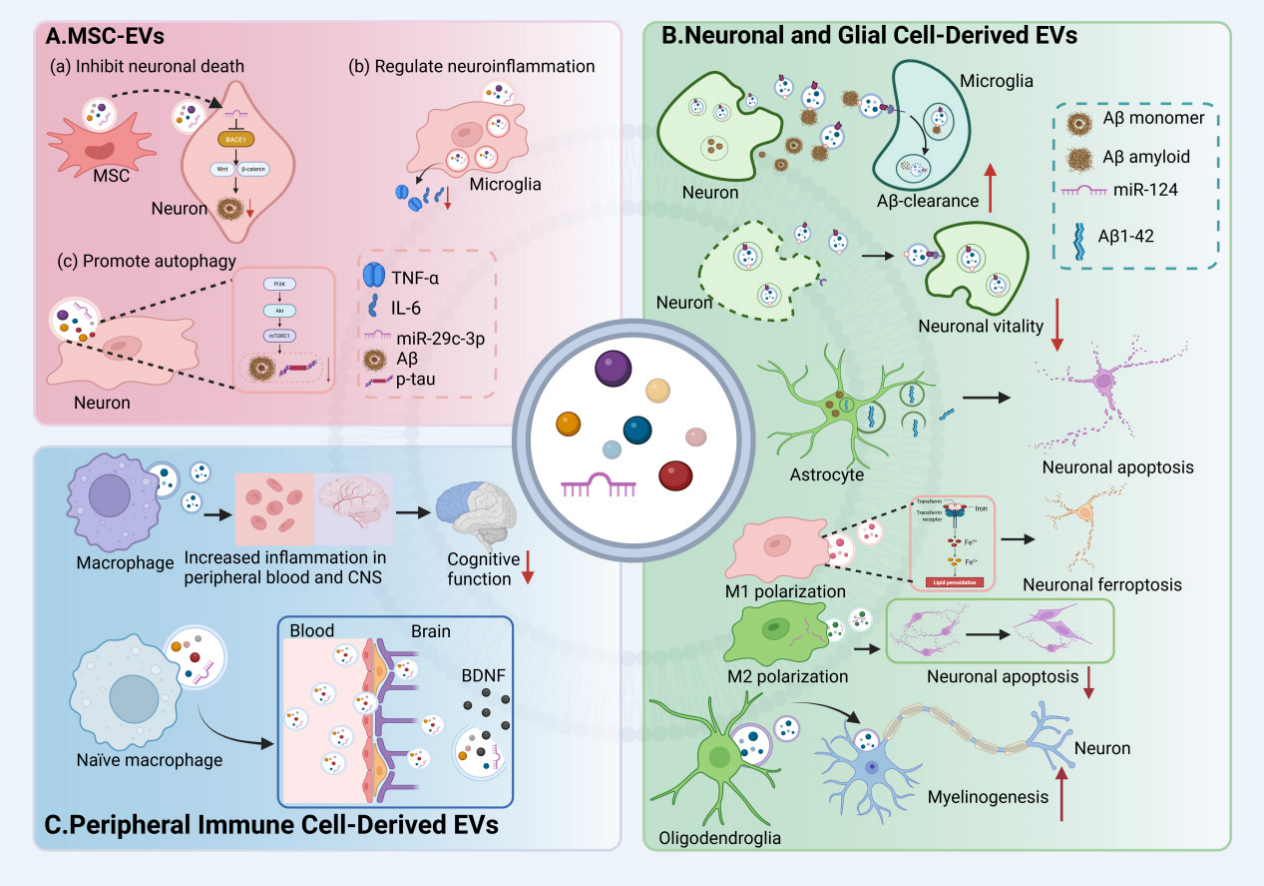
Bidirectional effects of EVs from different cellular sources in the central nervous system. (A) MSC-EVs inhibit neuronal death, modulate neuroinflammation, and regulate autophagy; (B) Neuronal EVs promote the clearance of Aβ, but may also inhibit neuronal viability. Astrocyte-derived EVs induce apoptosis in surrounding neurons. EVs derived from microglia exhibit a dual role, exacerbating neuronal injury while also reducing neuronal apoptosis; (C) EVs from macrophages promote the onset of neuroinflammation. Meanwhile, EVs from naïve macrophages are capable of delivering neurotrophic factors to the brain, exerting neuroprotective effects. MSC: Mesenchymal stem cell; EV: extracellular vesicle; TNF-α: tumor necrosis factor alpha; IL-6: Interleukin-6; Aβ: amyloid-beta; BDNF: brain-derived neurotrophic factor; CNS: central nervous system. Created in BioRender. yinghan, h. (2025) https://BioRender.com/c2ebp43

Thus, peripheral immune cell-derived EVs serve a dual role as both conveyors of pathological signals and potential therapeutic agents, modulating the central inflammatory response in AD and opening new perspectives for comprehensive treatment strategies [Figure 2].

## PROTEOMIC APPLICATIONS AND FUTURE PROSPECTS IN EVS RESEARCH FOR AD

As pivotal mediators of intercellular communication, the biological functions and clinical translation potential of EVs are largely determined by their cargo of bioactive molecules. Proteins serve as critical executors of EV biogenesis, targeting, and membrane fusion. More importantly, the specific integrin profiles, receptor tyrosine kinase activities, and diverse post-translational modifications carried by EVs collectively constitute a molecular “fingerprint” that reflects their cellular origin and pathophysiological status^[78]^. Therefore, proteomics stands as an indispensable tool for deciphering the biological functions and clinical value of EVs.

### Proteomic biomarkers revealed by proteomics

Proteomic profiling of EVs, particularly those derived from plasma, cerebrospinal fluid (CSF), urine, and other biofluids, has emerged as a powerful approach to identify novel biomarkers for AD. EVs, as nanoscale vesicles secreted by various cell types, carry cargo proteins reflecting the physiological and pathological state of their cells of origin, thus serving as a “liquid biopsy” for brain disorders.

Multiple studies utilizing mass spectrometry-based proteomics have successfully characterized the protein content of plasma-derived EVs, revealing distinct protein signatures that differentiate AD patients from healthy controls with high accuracy. For instance, a comprehensive proteomic analysis quantified 328 proteins in plasma-derived EVs, identifying a panel of six proteins (including Ig-like domain-containing protein, complement components C1q and C9, platelet glycoprotein Ib beta chain, Ras suppressor protein 1, and disintegrin and metalloproteinase domain 10) that robustly discriminate AD patients and correlate with cognitive performance^[19]^. Similarly, neuron-derived EVs isolated from plasma have shown increased levels of hemoglobin subunits in AD patients, suggesting their utility as diagnostic biomarkers^[79]^. Urine-derived EVs have also been profiled in AD mouse models, revealing early alterations in proteins such as Annexin 2 and Clusterin prior to Aβ plaque deposition, highlighting their potential for non-invasive early detection^[80]^. Proteomic studies of EVs from cerebrospinal fluid have demonstrated that proteases such as cathepsin B are associated with Aβ levels and disease progression in AD ^[81]^. Moreover, proteomic analyses have identified differentially expressed proteins related to immune response, synaptic function, and neuroinflammation within EVs, reflecting the multifaceted pathophysiology of AD^[82-83]^.

The integration of proteomic data with clinical parameters has enabled the development of diagnostic models with high sensitivity and specificity, facilitating early diagnosis and monitoring of disease progression. For example, plasma EV proteins have been shown to correlate with CSF biomarkers, brain imaging changes, and cognitive scores in early-onset mild cognitive impairment (a prodromal stage of AD)^[84]^. Additionally, machine learning approaches applied to proteomic datasets have identified biomarker panels capable of distinguishing early-stage AD patients from healthy individuals with approximately 79% accuracy^[85]^. Proteomic analysis of EVs offers a highly promising avenue for the discovery of novel biomarkers for AD, poised to revolutionize early diagnosis, treatment monitoring, and precision medicine in the field of neurodegenerative disorders.

### Changes in EV protein profiles at different stages of AD

Understanding the dynamic alterations in EV protein expression across different stages of AD is crucial for elucidating the molecular mechanisms underlying disease progression and for developing stage-specific diagnostics and therapeutic interventions.

Comparative analyses of MSC-EVs from pre-symptomatic (asymptomatic) and symptomatic AD patients have revealed distinct protein signatures that correlate with the pathological evolution of AD. The evolution of these EV protein signatures mirrors the clinical and pathological progression of AD. Critically, key alterations emerge years before clinical symptoms become apparent. For instance, reductions in synaptic proteins including growth-associated protein 43 (GAP43), neurogranin, synaptosome-associated protein 25 (SNAP25), and synaptotagmin 1 in EVs from symptomatic AD patients are detectable 5 to 7 years prior to overt cognitive impairment, serving as a vanguard of synaptic dysfunction^[86]^. In the very early, pre-symptomatic phases, such as subjective cognitive decline, EVs in peripheral blood dually labeled with NCAM and amphiphysin 1 already carry elevated levels of miR-29c-3p, underscoring their diagnostic value in prodromal phases^[87]^. This early dysregulation extends to proteins involved in Aβ metabolism and clearance, including clusterin, apolipoprotein E (ApoE), neprilysin, and angiotensin-converting enzyme, which are altered in urine-derived EVs from amyloid precursor protein (APP) transgenic mouse models^[88]^. As the disease advances to mild cognitive impairment, proteomic profiling of plasma-derived EVs reveals protein signatures that not only correlate with amyloid pathology and cognitive decline but can also distinguish amyloid-positive individuals, highlighting their pre-screening value^[84]^.

Overall, as AD progresses from the asymptomatic stage to the symptomatic stage, the composition of EV proteins undergoes dynamic changes, which not only reflects potential neuro-pathological changes but also offers hope for the early diagnosis of AD, monitoring of disease progression, and the development of targeted therapies.

### Proteomics-guided personalized therapy

The complexity of AD pathology, involving heterogeneous protein accumulations and multifactorial mechanisms, necessitates personalized therapeutic strategies that proteomics can uniquely support. For instance, proteomics enables the personalized identification of disease-specific biomarkers and therapeutic targets. Advanced mass spectrometry and bioinformatics analyses allow for comprehensive profiling of the protein cargo within EVs derived from various cell sources, which facilitates the identification of personalized, disease-specific biomarkers and therapeutic targets. Studies have identified distinct protein signatures in EVs from AD patients’ biofluids, such as serum and cerebrospinal fluid, highlighting candidate molecules such as alpha-1-antichymotrypsin (AACT) and C4b-binding protein alpha chain (C4BPα) that bind Aβ and may influence disease progression^[89]^. This proteomic insight paves the way for developing personalized, EV-based strategies, such as loading EVs with therapeutic proteins or engineering them for targeted drug delivery, thereby significantly advancing treatment precision and personalization.

In EV-based delivery systems, proteomic analysis further enables personalized therapeutics by ensuring the quality and stability of EV preparations and by identifying proteins associated with targeted delivery and therapeutic payloads. For example, proteomic characterization of EVs from different stem cell sources reveals unique protein compositions that may influence their regenerative potential and immunomodulatory effects^[90]^. This analytical approach allows for the selection of the most suitable EV source based on individual patient characteristics. For instance, specific proteins in MSC-EVs, particularly those involved in autophagy, contribute to neuroprotection. Furthermore, EVs from hypoxia-preconditioned MSCs can enhance host cell migration and tissue repair, potentially offering a personalized strategy for neural regeneration^[91]^. Additionally, EVs released from human induced pluripotent stem cell (iPSC)-derived neurons expressing the AD familial A246E mutant form of presenilin 1 (mPS1) exhibit aberrant kinase and phosphatase profiles indicative of dysregulated tau protein phosphorylation. This finding suggests that interventions targeting specific genetic mutations can restore this imbalance, underscoring a highly tailored therapeutic potential^[92]^.

Proteomics further advances personalized therapeutic decisions by elucidating disease mechanisms and sources of treatment resistance. In cancer research, the proteomic signatures of EVs have been utilized to guide personalized treatment strategies^[93-94]^. Proteomics also informs the assessment of individual patient treatment efficacy by monitoring changes in protein expression and signaling pathways, such as synaptogenesis and neurovascular coupling, which are critical for cognitive function and are modulated by therapeutic agents^[95]^. Additionally, serum-derived EVs from AD patients showed altered protein content related to energy metabolism, oxidative stress, and neuroinflammation, which could be modulated by therapeutic agents (e.g., ferulic acid), thereby informing the development of tailored regulatory regimens for different patients^[82,96]^.

Furthermore, the integration of proteomic data with other omics datasets is central to realizing personalized medicine. By combining information from genomics, metabolomics, and microbiomics, fully individualized therapeutic strategies can be devised based on the unique characteristics of each person. A prime example of this multimodal approach in clinical trials is the Cognitive Coaching in Alzheimer’s (COCOA) trial. This study achieved a shift from a “static protocol” to a “dynamic intervention” by continuously collecting and integrating participants’ multi-omics and clinical data. Specifically, it established a personalized management plan for each patient, one that allows for real-time feedback and adjustments based on periodic monitoring data, such as proteomic changes, thereby enabling a precise and effective delay in cognitive decline^[97-98]^.

Taken together, proteomics-guided optimization of EV-based therapies constitutes a cornerstone of personalized medicine for AD. By identifying disease-specific protein biomarkers, monitoring therapeutic responses, and customizing EV cargo, proteomic technologies significantly enhance the safety and efficacy of EV-based therapies. With ongoing breakthroughs in high-throughput proteomics and its deepening integration with artificial intelligence, personalized therapeutic strategies will be further refined, ultimately yielding more precise and favorable clinical outcomes for patients with AD.

## BENCH-TO-BEDSIDE INVESTIGATION OF EVS IN AD

### Therapeutic efficacy validation in animal models

In various animal models of AD, EVs have demonstrated significant therapeutic potential, being able to improve the cognitive functions of the model animals. For instance, intracerebroventricular injection of bone marrow mesenchymal stem cell (BMSC)-derived EVs significantly improved behavioral performance in streptozotocin (STZ)-induced AD mice across a battery of tests, including the open field test (OFT), elevated plus maze (EPM), novel object recognition test (NORT), Y-maze, and tail suspension test (TST)^[99]^. Similarly, intranasal administration of human MSC-derived EVs (hMSC-EVs) enhanced both spatial and non-spatial learning and memory in five-times familial Alzheimer’s disease (5×FAD) mice, as evaluated by the NORT, Y-maze, and Barnes maze^[100]^. Consistent with these findings, amyloid precursor protein/presenilin 1 (APP/PS1) double transgenic mice treated with MSC-EVs exhibited significantly improved learning and memory, evidenced by superior performance in the Morris water maze^[101]^. Furthermore, treatment of 5×FAD mice with hippocampal neuron-derived EVs effectively mitigated cognitive decline, as assessed by the Rotarod test for motor coordination, OFT for locomotor and exploratory activity, and contextual fear conditioning for memory^[22]^. EVs derived from M2-polarized microglia have been shown to improve learning and memory in both 5×FAD mice and an AD model induced by intracerebroventricular Aβ1−42 injection, with efficacy demonstrated in the NORT, Morris water maze, Y-maze, and OFT^[102-103]^.

### Molecular mechanisms and signaling pathways mediated by EVs

#### Energy metabolism and mitochondrial function regulation

Mitochondrial dysfunction, manifested as impaired oxidative phosphorylation^[104]^, disrupted energy metabolism^[105]^, defective mitophagy^[106]^, and mutations in mitochondrial DNA^[107]^, is a core pathological feature of AD. It is also a critical factor driving neuronal failure and cognitive decline^[108]^. Acting as key delivery vehicles, EVs can transport metabolic proteins, enzymes, and regulatory molecules to effectively restore mitochondrial homeostasis in the brain by reshaping cellular energy metabolism mechanisms^[109]^.

The therapeutic potential of EVs stems from their ability to deliver key bioactive molecules that regulate mitochondrial function, thereby improving neuronal energy metabolism and enhancing cellular resistance to stress. For example, EVs can not only mediate the activation of nuclear factor (erythroid-derived 2)-like 2 (Nrf2) to enhance mitochondrial antioxidant capacity, but also support neuronal survival under stress by regulating hypoxia-inducible factor 1 (HIF1)-dependent metabolic reprogramming^[110]^. EVs targeting neuronal mitochondria protect neurons from apoptosis and synaptic loss by inhibiting oxidative stress in mitochondrial-dependent mechanisms, thereby improving the mechanism of cognitive decline in AD^[111]^.

EV-mediated mitochondrial regulation also influences the energy metabolism of microglia. The translocator protein (TSPO) and hexokinase-2 (HK2), components of mitochondrial complexes, govern the metabolic switch between oxidative phosphorylation and glycolysis in microglia, affecting their phagocytic capacity to clear toxic aggregates in AD^[112]^. Restoring the function of these proteins via EV delivery holds promise for improving mitochondrial function, influencing the energy metabolism of microglia, and promoting neurogenesis.

In conclusion, EVs can effectively address the core pathological issue of energy metabolism disorder in AD. The potential of multi-target intervention based on EVs lies in simultaneously enhancing mitochondrial function and reducing oxidative stress, which is expected to prevent and reverse the biological energy depletion that leads to neuronal dysfunction.

#### Protein homeostasis and autophagy-lysosomal pathway

The autophagy-lysosomal pathway (ALP) is a critical proteostatic system for degrading damaged organelles and aggregated proteins. ALP dysfunction represents a core pathological event in AD, driven by a combination of genetic risk factors and the aging process.

Apolipoprotein E4 (APOE 4), a major genetic risk factor for AD, disrupts endosome-lysosome and autophagy pathways, leading to impaired autophagy initiation, lysosomal dysfunction, and subsequent Aβ and tau accumulation. APOE4-mediated dysregulation promotes lysosomal stress and defective vesicle trafficking, contributing to neuronal vulnerability^[113-114]^. This age-related decline is compounded by dysregulation in other degradation systems, as exemplified by Ras-related protein Rab-21 (Rab21), a GTPase processed by both the ALP and the ubiquitin-proteasome system^[115]^. Astrocyte autophagy also modulates neuroinflammation and protein aggregation, influencing AD progression and representing another cellular target to restore proteostasis^[116]^. The transcription factor EB (TFEB), a master regulator of autophagy and lysosomal biogenesis, is frequently dysfunctional in AD. Studies demonstrate that activating TFEB enhances microglial clearance of Aβ and improves cognitive function in AD models, underscoring its therapeutic potential^[117-118]^. BMSC-EVs can inhibit mTOR, thereby promoting TFEB nuclear translocation and restoring lysosomal function, which alleviates ALP dysfunction^[119]^. Similarly, EVs derived from retinal pigment epithelial cells have been shown to influence the ALP by modulating lysosomal function^[120]^.

EVs participate in the intercellular transfer of misfolded proteins, including Aβ and tau, “which can be regulated by autophagy cargo receptors such as sequestosome 1 (Sqstm1) and optineurin. These receptors inhibit EVs secretion by promoting autophagic degradation of ubiquitinated cargos, whereas dysregulation leads to increased pathogenic protein spread via EVs^[121]^. Targeting this mechanism holds promise for curbing the adverse effects mediated by EVs.

#### Inflammatory response and immune regulation

In AD, neuroinflammation and immunity dysregulation are critical pathological features contributing to neuronal damage and disease progression. Glial and stem cell-derived EVs play distinct yet complementary roles in regulating inflammation and immune. Astrocyte-derived EVs are enriched with growth factors and proteins supporting metabolic and ubiquitin-dependent protein balance. Notably, miR-146a is upregulated in astrocytes and their EVs in response to inflammatory stimuli, subsequently regulating key signaling pathways such as nuclear factor kappa-B (NF-κB) and Janus kinase/signal transducer and activator of transcription (JAK/STAT) to control cytokine production and immune activation^[122,123]^. In contrast, microglia-derived EVs are more closely associated with immune and oxidative stress pathways^[124]^. Beyond resident glial cells, MSC-EVs exhibit potent immunomodulatory effects. They transfer nucleic acids, proteins, and lipids that attenuate microglial activation, reduce pro-inflammatory cytokine release, and promote a shift toward an anti-inflammatory phenotype^[53,125]^. Specifically, bone marrow MSC-EVs have been shown to inhibit the activation of both microglia and astrocytes and their associated neuroinflammation, thereby alleviating cognitive decline in AD-like mouse models^[99]^.

Additionally, EVs can cross the BBB and mediate crosstalk between peripheral immune signals and CNS immune cells, further influencing neuroinflammatory responses^[123]^. Interestingly, bacterial outer membrane vesicles (OMVs) from Helicobacter pylori have been implicated in triggering neuroinflammation by activating microglia and astrocytes, suggesting that EVs from peripheral sources may exacerbate CNS inflammation in AD^[126]^.

### Early clinical studies

Recent advances in the study of EVs, particularly MSC-EVs, have propelled these EV-based therapies into the forefront of AD research, with some formulations progressing into preclinical and early-phase clinical trials.

Biomarker studies utilizing neuron-derived EVs isolated from the peripheral blood of AD patients suggest that these markers hold potential for the early diagnosis and monitoring of AD, which is crucial for timely therapeutic intervention^[38]^. EV-associated biomarkers, including Aβ1-42, phosphorylated tau species, and neuroinflammatory markers, whose levels correlate with disease stages from preclinical to clinical AD, providing surrogate endpoints for clinical trials^[127]^. In parallel, early clinical investigations have begun to evaluate the safety and preliminary efficacy of EV-based therapies. For instance, a nanoparticle formulation based on EVs and carrying small interfering RNA (siRNA) targeting the oncogenic mutant Kirsten rat sarcoma viral oncogene homolog (Kras) gene demonstrated favorable tolerability and safety in a Phase I trial, with no dose-limiting toxicities observed; it also remodeled the tumor immune microenvironment and promoted anti-tumor immune responses^[128]^. Similarly, a Phase I trial of human umbilical cord-derived MSC-EVs for pulmonary fibrosis confirmed the safety and efficacy of EV-based treatment^[129]^. Although clinical trials of EV-based therapy for AD remain limited, an early study in patients with mild-to-moderate AD suggested that adipose MSC-EVs were safe and potentially effective. No significant immune reactions or carcinogenic risks were detected, underscoring the inherently low-risk profile of EV-based therapeutics^[130]^.

Despite these encouraging developments, several challenges remain before EV therapies can be widely adopted in clinical practice. These include standardization of EV isolation and characterization methods, optimization of dosing and delivery routes, large-scale manufacturing, and regulatory considerations. Innovative approaches such as engineering EVs to enhance targeting and therapeutic payload delivery are under investigation to improve efficacy^[131-132]^. Furthermore, the heterogeneity of EV populations and complexity of their cargo necessitate rigorous quality control to ensure reproducibility and safety. Moving forward, robust preclinical evidence will be essential to fully validate the efficacy and safety of EV-based therapies as a novel strategy for AD treatment.

## CHALLENGES AND PROSPECTS OF EV THERAPY

### Challenges in EV purification and standardization

The clinical translation of EVs is significantly hampered by challenges in purification and standardization. Current isolation methods often fail to produce EV preparations that are both highly pure and compositionally uniform - essential attributes for ensuring reproducibility and safety in clinical use. Traditional isolation techniques such as ultracentrifugation, size exclusion chromatography (SEC), polymer precipitation, and immunoaffinity capture each have inherent limitations. Ultracentrifugation, widely regarded as the gold standard, often co-isolates contaminating proteins, small microvesicles, and apoptotic bodies, leading to heterogeneous preparations with variable purity^[133-134]^. For instance, ultracentrifugation alone was shown to result in contamination by non-EV proteins such as calnexin, whereas combining ultracentrifugation with SEC significantly enhanced purity by effectively removing such contaminants^[133]^. SEC improves purity but can be labor-intensive and limited by low throughput. Polymer-based precipitation methods provide higher yields but often compromise purity due to co-precipitation of non-vesicular components^[135]^. Immunoaffinity-based methods offer specificity by targeting EV surface markers (e.g., CD63, CD9, CD81) but are constrained by cost, scalability, and potential alteration of vesicle integrity^[136]^. To address these limitations, innovative approaches such as gold nanoparticle-assisted low-speed centrifugation have been developed, enabling efficient isolation of small EVs with significantly reduced processing time and equipment requirements^[84]^ [Table 1].

**Table 1:** Advantages and disadvantages of exosome separation technology

**Methods**	**Advantages**	**Disadvantages**	**References**
Ultracentrifugation	1. Gold standard 2. Label-free 3. Large sample volume 4. Broad applicability	1. Expensive equipment 2. Time-consuming 3. Low yield and heterogeneity 4. Potential vesicle damage	[137]
Size exclusion chromatography	1. High purity 2. Gentle conditions 3. Good reproducibility 4. Suitable for complex samples	1. Sample dilution 2. Limited loading capacity 3. Resolution limits 4. Column clogging risk	[138]
Polymer precipitation	1. Simple operation 2. Fast and convenient 3. High yield 4. High-throughput friendly	1. Very low purity 2. May interfere with downstream assays 3. Requires washing steps	[139]
Immunoaffinity capture	1. High specificity 2. High purity 3. Suitable for low-concentration samples.	1. High cost 2. Not universal 3. Harsh elution	[136]

EV heterogeneity is influenced by cellular origin, isolation method, and biofluid source, with differences in EV protein composition directly affecting biological activity and therapeutic efficacy^[140]^. This variability underscores the urgent need for standardized protocols and quality control measures, including robust characterization techniques such as nanoparticle tracking analysis, electron microscopy, flow cytometry, and proteomic profiling^[141]^.

### Safety and long-term effect evaluation

The safety profile and long-term effects of EV-based therapies remain critical considerations for their clinical translation. MSC-EVs have demonstrated promising therapeutic potential due to their immunomodulatory, regenerative, and paracrine capabilities while circumventing many risks associated with cell-based therapies, such as immune rejection and tumorigenicity. Several studies underscore that MSC-EVs are generally well-tolerated and exhibit lower immunogenicity compared to their parent cells, enabling safer systemic administration with minimal adverse immune responses^[142-144]^. Moreover, clinical trials employing adipose stem cell-derived EVs in dermatological applications reported mild side effects such as transient erythema but no serious adverse events, indicating favorable safety profiles in humans^[145]^. It is worth noting that the potential of EVs in sports medicine to promote tissue repair, enhance muscle function, and even improve athletic performance is attracting attention from outside the field^[146-147]^. Reports indicate that some athletes have begun attempting to use EV injections to accelerate injury recovery or enhance competitive performance. However, such applications operate entirely outside the formal clinical research and development system. Their safety, efficacy, and long-term side effects lack rigorous scientific evaluation, and the source, purity, and dosage of the products used are not guaranteed.

The immunomodulatory properties of EVs present a dual-edged sword; while beneficial, they risk causing unintended immunosuppression or aberrant inflammatory responses, especially in vulnerable populations, and require careful monitoring. A preclinical meta-analysis focusing on diabetic peripheral neuropathy models confirmed that EV-based therapy improved neurovascular remodeling without detectable toxicity. However, it also emphasized the necessity for larger, well-designed preclinical trials to definitively validate their long-term safety^[148]^.

Current evidence suggests that EV-based therapies possess a favorable safety profile with minimal immunogenicity and toxicity, making them attractive candidates for regenerative medicine. Nonetheless, comprehensive long-term safety assessments, including immune response profiling, biodistribution, and potential off-target effects, are imperative. Future research should prioritize multicenter, long-term longitudinal clinical trials to fully elucidate their safety and therapeutic durability in AD and other chronic conditions.

## TRANSLATION PATH OF EV THERAPY

EV-based therapy faces numerous challenges during clinical translation, including large-scale production under high-quality Good Manufacturing Practice (GMP) standards, complex regulatory policies, and multiple considerations in clinical trial design.

### GMP production process

As an emerging biological therapeutic product, EV preparations are gradually entering the clinical application stage, with their production and quality control subject to stringent regulations and standards. The production of EVs in GMP facilities is established with reference to the "Guideline on Quality, Non-clinical and Clinical Assessment of EV Therapy Products" Guidelines for Quality, Non-Clinical, and Clinical Evaluation of Extracellular Vesicle Therapeutic Products issued by the Korean Ministry of Food and Drug Safety (KMFDS). This guideline, the first of its kind globally, covers standardized requirements for the entire process, from cell source selection, cell culture, EV isolation and purification, quality testing, to final product storage and transportation^[149]^.

First, during the cell culture stage, appropriate cell sources must be selected, such as MSCs or other rigorously screened and quality-controlled cell lines. Cell culture should utilize serum-free media or serum from which EVs have been removed via ultracentrifugation to prevent contamination by exogenous proteins and EVs ensuring the purity and safety of the subsequent products^[150]^. However, another study has shown that while the use of serum-free media increases the quantity of EVs produced, it also alters their protein composition^[151]^. Therefore, in practical production, the choice between serum-free or processed standard media requires careful consideration based on the specific quality attributes of the target product. Additionally, applying three-dimensional dynamic culture systems can achieve large-scale production of EVs and enhance their yield^[152]^.

Second, innovative methods continue to emerge in the collection and purification of EVs. Biosensor-based separation platforms combined with microfluidic technology enable single-sample input and output, reducing cross-contamination risks and providing higher separation efficiency and quantitative sensitivity^[153]^. Novel materials, such as dual-function composite magnetic nanomaterials modified with aptamers and metal oxides, have demonstrated enhanced EV enrichment capabilities and selectivity by combining ligand specificity with phospholipid bilayer affinity^[154]^. Tangential flow filtration (TFF) is widely used for large-scale purification, preserving EV integrity, improving purification efficiency and reproducibility, and meeting the quality requirements for clinical-grade products^[155]^. Gold nanoparticle-assisted low-speed centrifugation methods enable efficient isolation of small EVs while significantly reducing processing time and equipment requirements^[84]^. The application of automation and large-scale production technologies is key to achieving clinical translation of EVs. Using stirred-tank reactors (STRs) in combination with microcarrier culture for efficient cell expansion and continuous EV collection has been shown to stably produce high-purity MSC-derived EVs in compliance with GMP standards^[156]^. Furthermore, automated closed systems can minimize human operational errors and contamination risks, enhancing product consistency and safety^[157]^. Notably, EVs must not only carry neuroprotective factors but also avoid propagating pathological proteins such as β-amyloid and tau proteins, imposing higher demands on isolation technologies^[158-159]^.

Purified EVs require comprehensive quality control to ensure their critical quality attributes meet predefined standards. This includes characterizing the particle size distribution, verifying the presence of specific protein markers such as CD9, CD63, CD81, and syntenin-1, assessing morphological integrity, and evaluating biological activity^[156,160]^. Additionally, strict adherence to GMP is essential, encompassing aseptic handling and validated sterilization procedures. Prior to release, the final product must undergo bacterial endotoxin testing using methods such as the Limulus amebocyte lysate assay, along with microbial limit testing^[161]^. Additionally, assessing the storage stability and physical stability of EVs is critical for ensuring safety. Appropriate storage conditions can prevent EV aggregation and functional loss. Studies indicate that cryopreservation is currently a common method for maintaining EV stability^[162]^. Furthermore, the application of lyophilization technology is being explored to enhance long-term storage stability^[163]^. It is important to note that the composition of the storage buffer also affects EV stability^[164]^. Therefore, careful consideration of the source and type of EVs, storage conditions, and intended downstream applications is essential to ensure optimal preservation while avoiding toxicity.

### Regulatory policies

The regulatory framework for EV-based therapy is continuously evolving, with relevant agencies emphasizing the need to establish standardized characterization and production protocols to ensure product safety and efficacy^[165]^. Currently, regulatory approaches for EV-based products vary globally, primarily divided into two paths based on how regulatory agencies define the nature of the product. One regulatory approach focuses on the intrinsic components of EVs and their modulatory effects on human physiological processes, a path primarily adopted by drug regulatory agencies in the United States and Europe. Under this framework, the active ingredient of the product is the core of regulatory identification, while the preparation process is of secondary importance. Another regulatory logic, exemplified by Japan and Korea, emphasizes the extraction methods and cellular origins of EVs. In this system, EVs prepared from the same source cells using consistent processes are classified as the same type of drug, with their functions primarily defined based on the original roles of the parent cells^[166]^.

In terms of specific regional regulatory practices, the United States defines EV-based products as biologics, requiring submission through the Investigational New Drug (IND) application process and undergoing rigorous preclinical and quality control reviews by the US Food and Drug Administration (USFDA)^[167]^. In the European Union, such products are regulated under the Advanced Therapy Medicinal Products (ATMP) framework, subject to centralized review by the European Medicines Agency (EMEA) and its expert committees, with specific guidelines applicable to gene therapies and similar products^[168]^. In Japan, EVs devoid of living cell components are primarily regulated as conventional drugs, with different regulatory systems applying based on whether they contain living cell components^[169]^.

Despite varying regulatory paths, there is a growing consensus among countries on the importance of GMP compliance and full-process quality control, which may facilitate international harmonization of regulatory standards, thereby improving global review efficiency and product accessibility. However, the regulation of EV-based products still faces significant challenges. First, the heterogeneity and complex biological characteristics of EVs make standardized production and quality control extremely difficult, with a lack of uniform testing methods and standardized indicators. Second, insufficient preclinical and clinical data limits comprehensive evaluation of EV safety and efficacy, increasing regulatory uncertainty. Additionally, differences in cross-border regulatory policies pose obstacles to international cooperation and global product promotion. Consequently, there is a general consensus within the industry that establishing a unified risk classification framework, clarifying the regulatory positioning of EV-based products, and promoting the development of internationally accepted quality standards and testing methods are key to advancing the standardization and clinical application of EV-based therapies.

### Clinical trial design

For clinical trial design of EV-based therapy in AD, key considerations include participant selection criteria, dose standardization and escalation protocols, and safety monitoring. First, participant selection should clearly define diagnostic criteria for the disease, including both early- and mid-to-late-stage AD patients, while excluding those with severe comorbidities or immune system abnormalities to ensure consistency and safety. Notably, emerging biomarkers, such as specific proteins and RNAs in salivary EVs, are being explored for early identification and subtype stratification of AD, offering new tools for more precise participant enrollment in the future^[170]^. Due to the diversity of EVs sources and variations in production processes and purity, establishing unified dosing units and administration regimens is particularly important. Currently, dosing is often based on total particle number. Dose escalation typically follows the classic “3 + 3” design, starting from extremely low doses and gradually increasing to higher doses^[130]^. Throughout this process, pharmacokinetic studies must be incorporated to clarify the distribution, metabolism, and clearance of EVs *in vivo*, while simultaneously monitoring dynamic changes in pharmacodynamic biomarkers to scientifically determine the optimal therapeutic dose window.

Although EVs are generally considered to have low immunogenicity, their long-term safety requires careful evaluation. Some studies indicate that engineered modifications do not significantly increase the risk of adverse events^[171]^. However, theoretical risks of immune activation remain, particularly in long-term trials involving multiple administrations. Therefore, safety monitoring mechanisms should extend beyond routine vital signs and laboratory tests to include targeted immunological monitoring, such as analysis of specific cytokine profiles and screening for autoantibodies, to early detect potential immune-inflammatory responses. These design details are crucial for enhancing the success rate of clinical translation of EV therapy for AD.

Multicenter clinical trials are increasingly important in EV-based therapy research for AD due to their ability to increase sample size and result representativeness. Multicenter trials require establishing unified coordination mechanisms to ensure all centers adhere to consistent standard operating procedures (SOPs) in participant recruitment, treatment implementation, data collection, and safety monitoring. Regarding data management, centralized electronic data capture (EDC) systems should be employed to enable real-time data upload, quality control, and secure storage, ensuring data integrity and traceability. The clinical translation process necessitates rigorous safety assessments and well-designed clinical trials. Effective multicenter coordination and scientific data management not only enhance trial quality but also provide a solid foundation for subsequent drug registration and clinical application.

### Translation pathway recommendations and technology roadmap

During the laboratory research phase, efforts should focus on optimizing EV source selection, extraction, and purification techniques. In the preclinical validation phase, *in vitro* cell models and animal models should be systematically used to evaluate the safety, pharmacokinetics, and therapeutic efficacy of EVs. During the clinical trial phase, a stepwise approach is recommended, initially focusing on safety and dose escalation, gradually expanding to efficacy evaluation. Given the heterogeneity of AD, clinical trials should incorporate personalized design strategies, exploring precision treatment approaches for patient groups with different disease stages and pathological characteristics. In the commercial production phase, it is essential to establish a GMP-compliant, large-scale production system to ensure batch-to-batch consistency and quality safety of EV-based products. Simultaneously, a comprehensive cold chain logistics and storage system must be established to maintain product stability and activity. Furthermore, to facilitate the smooth implementation of the above translation pathway, it is advisable to establish interdisciplinary collaboration platforms integrating experts from fields such as stem cell biology, nanotechnology, pharmaceutical engineering, clinical medicine, and regulatory policy. Through multidisciplinary collaboration, technological integration and innovation can be promoted, accelerating the clinical translation process of EV-based therapy for AD.

Standardized GMP requirements, international harmonization of regulatory policies, innovative clinical trial designs, and real-world evidence will significantly advance the commercialization of EV-based therapeutic products. In the future, with the gradual improvement of regulatory systems and the maturation of the industry chain, EV-based therapy for AD is expected to become a key strategy for overcoming traditional therapeutic limitations, ushering in a new era of precision and personalized AD treatment.

## EV ENGINEERING STRATEGIES

### EV engineering process

While EV-based therapies offer multiple advantages, naturally occurring EVs as therapeutic carriers often face challenges such as insufficient targeting specificity. To address these limitations, a series of engineering strategies have been developed. The feasibility and considerable potential of these approaches have been extensively validated in oncology research and are beginning to show promise in the field of AD as well. The engineering of EVs is a systematic and intricately interconnected process aimed at transforming natural exosomes into an intelligent delivery system that combines high targeting specificity, high drug-loading capacity, and controlled release. The entire procedure can be systematically divided into three stages: upstream preparation, midstream engineering, and downstream validation.

The upstream preparation stage begins with the genetic modification of parent cells, which is crucial for determining the innate properties of the EVs. Specific expression vectors are constructed using molecular cloning techniques. For example, to produce EVs that specifically target tumor cells with high levels of the epidermal growth factor receptor (EGFR), the sequence for the targeting peptide GE11 can be fused to the gene for the transmembrane protein CD63. This forms a plasmid or lentiviral vector, which is then introduced into cells such as human embryonic kidney 293T (HEK293T) cells. After screening, engineered cell lines that stably secrete EVs displaying the GE11 peptide on their surface are obtained. These engineered cells are expanded in culture medium, and their conditioned supernatant becomes enriched with targeting shell EVs that have not yet been loaded. Subsequently, highly pure, basic EVs are isolated from the supernatant, laying the foundation for subsequent loading^[172]^.

The midstream engineering stage, which serves as the core process for endowing EVs with therapeutic functions, involves two key steps: drug loading and secondary targeting modification. These steps can be performed either independently or in cooperation. The drug loading strategy must be tailored to the properties of the cargo. For small-molecule drugs such as curcumin, simple co-incubation is often used, relying on concentration gradients for passive loading into the lipid bilayer^[173]^; in contrast, for macromolecules or charged molecules (e.g., siRNA), physical methods such as electroporation or sonication are required to transiently disrupt the membrane structure and improve loading efficiency^[174-175]^. Concurrently or subsequently, more precise targeting upgrades can be performed on purified EVs. In addition to the aforementioned methods, protein ligase-mediated covalent conjugation has emerged as a novel strategy. For example, using protein ligases to efficiently conjugate EGFR-targeting peptides or nanobodies onto the surface of EVs has been shown to significantly enhance their enrichment in EGFR-positive tumor cells in both *in vitro* and *in vivo* experiments, and to markedly improve the tumor-inhibiting effect of low-dose paclitaxel^[176]^.

The downstream validation stage is the quality control checkpoint to ensure the safety and efficacy of engineered EVs, encompassing physical, biochemical, and functional aspects. First, nanoparticle tracking analysis is used to confirm the particle size distribution and concentration, and transmission electron microscopy is employed to observe their typical cup-shaped morphology. Second, Western Blot is performed to detect markers to verify their purity and identity. The most critical functional validation is then conducted in cellular and animal models: *in vitro*, it must be demonstrated that the EVs specifically bind to and are internalized by target cells, but not by other cell types; *in vivo*, techniques such as small animal live imaging are used to track their biodistribution, and ultimately, their actual therapeutic efficacy and safety are evaluated. For instance, a recent study fused EVs with drug-loaded liposomes; *in vivo* real-time imaging demonstrated that the targeted system achieved specific pulmonary accumulation in a pulmonary metastatic melanoma model^[177]^.

In summary, the engineering process for EVs is an interdisciplinary practice integrating molecular biology and nanotechnology. Through precise modular design, each modification step aims to optimize their delivery efficiency, with the ultimate goal of transforming them into a next-generation targeted therapeutic tool capable of overcoming the lack of targeting specificity of traditional drugs and reducing systemic side effects.

### Targeted modification and delivery system optimization

Although glioma and AD are pathologically distinct, they face similar challenges in intracerebral delivery when utilizing engineered EVs for treatment. Due to the urgent clinical need in glioma, a mature and proactive advanced technological system has been developed, whose design concepts hold significant reference value for AD research.

#### Targeting

In glioma therapy, EV targeting has evolved beyond basic BBB penetration to develop multiple active strategies, advancing to the specific recognition of tumor cells. For instance, the Angiopep-2 peptide is utilized to simultaneously target the low-density lipoprotein receptor-related protein 1, which is highly expressed on both the BBB and glioma cells. This approach is combined with the Transactivator of transcription peptide to enhance tumor tissue penetration, thereby constructing an efficient dual-targeting delivery system^[178]^. To enhance targeting efficiency, physical navigation was introduced. EVs conjugated with superparamagnetic iron oxide nanoparticles (SPIONs) achieve directed accumulation at the tumor site under the guidance of an external magnetic field^[179]^. An even more ingenious strategy is homotypic targeting, which directly utilizes EVs secreted by glioma cells themselves, capitalizing on their natural property of homing to parental cells as carriers. Combining these two strategies, by modifying glioma cell-derived EVs with SPIONs, achieves dual synergistic targeting through biological homing and physical magnetic navigation, significantly enhancing intracerebral delivery efficiency^[180]^.

In contrast, targeting strategies in the AD field remain primarily centered on basic brain targeting. For instance, surface modification of EVs with neuron-specific rabies virus glycoprotein (RVG) enables their specific binding to neurons, thereby increasing drug enrichment in AD-affected brain regions^[2]^. Furthermore, engineered EVs expressing somatostatin receptor-targeting ligands have also been demonstrated to achieve specific drug delivery to the hippocampal region^[181]^. In the future, EV-based therapies for AD could build on advanced approaches from glioma research, using physical-assisted strategies such as magnetic or ultrasound targeting to guide drug-loaded EVs to accumulate in early AD-affected brain regions, including the hippocampus and entorhinal cortex. Developing engineered EVs derived from microglia or neurons to leverage their endogenous homing properties could enable more precise delivery of drugs to target neural cell populations.

#### Release control

In terms of controlling drug release, engineered EVs for glioma have become highly sophisticated. Their key design is to use the unique microenvironment of the tumor as a trigger. These conditions include high glutathione levels, slight acidity, and high amounts of specific enzymes. An ingenious design involves integrating a reduction-responsive oligopeptide containing cysteine residues into EVs, enabling doxorubicin to be effectively locked within via disulfide cross-linking during systemic circulation. Once the EVs accumulate in glioblastoma tissues where glutathione levels are extremely high, the disulfide bonds are reductively cleaved, resulting in efficient drug release at the target site^[182]^. Beyond this, pH-sensitive methods are also common. These use chemical links that break in acidic environments, releasing the drug specifically in the slightly acidic tumor. One innovative study created an EV modified with transferrin using an acid-cleavable link. This system breaks apart in the acidic environment of lysosomes within BBB cells, helping drug-loaded EVs cross the barrier more efficiently^[183]^. Enzyme-based strategies are also well-developed. They use peptide links that can be cut by enzymes, such as matrix metalloproteinases, which are found in high amounts around tumors. This allows drug release exactly in regions with abnormal enzymatic activity^[184]^.

This intelligent release logic based on specific enzymes in the pathological microenvironment is beginning to emerge in AD therapy. Recent research constructed an enzyme-cleavable antibody-conjugated engineered EV, whose linker can be specifically cleaved by beta-secretase, a key enzyme in AD pathology. This holds promise for achieving precise, site-specific drug release in Aβ-related lesion areas^[185]^. This suggests that the AD field can more systematically adopt the environment-responsive concept from tumor therapy. The AD brain also possesses a unique pathological microenvironment, for example, significant oxidative stress leading to glutathione level imbalance. This provides a theoretical basis for designing disulfide-linked redox-responsive EVs that can specifically release drugs near neurons with abnormal redox states. Going a step further, future designs might use molecules that bind specifically to Aβ oligomers as “intelligent switches”, allowing EVs to release therapeutic cargo only when they encounter high concentrations of pathological protein aggregates, thereby achieving precise targeting of therapy.

#### Immune modulation

In the dimension of immune modulation, the objectives of the regulatory strategies for the two conditions are distinctly different. Glioma therapy aims to actively reverse its highly immunosuppressive microenvironment and has developed systematic offensive strategies. For example, engineering EVs to overexpress stimulator of interferon genes (STING) directly activates antigen-presenting cells within the tumor and the cyclic GMP-AMP synthase (cGAS)-STING pathway, initiating anti-tumor immune responses and inducing the formation of tertiary lymphoid structures with cytotoxic functions^[186]^. On the other hand, EVs surface-modified with targeting peptides and loaded with siRNA targeting programmed death ligand 1 (PD-L1) can synergize with radiotherapy to precisely silence immune checkpoint molecules, effectively relieving suppression of T cells^[187]^. These two strategies work from the directions of active activation and inhibition relief, respectively, aiming to transform the immunologically inert tumor microenvironment into an immunologically active battleground, showcasing the powerful regulatory capability of EVs as a multifunctional platform.

In contrast, the core objective of current neuroinflammation regulation strategies in the AD field leans more towards modulating the abnormally activated immune system. For instance, EVs derived from a mixture of MSCs and neutrophils can effectively target AD-related brain regions by leveraging the adhesion molecules lymphocyte function-associated antigen 1 (LFA-1)/intercellular adhesion molecule 1 (ICAM-1) on neutrophil surfaces. By inhibiting inflammation and increasing microglial abundance, they improve cognitive function in model mice^[188]^. Another study fused the natural brain-homing ability of brain microvascular endothelial cells with the inflammatory targeting properties of macrophages to construct hybrid EVs. These vesicles can efficiently penetrate the BBB, accumulate in brain inflammation regions, promote Aβ clearance, and alleviate microglial dysfunction^[189]^. Although these studies have made progress, their core logic still relies on utilizing the natural properties of EVs or simple modifications to regulate excessive inflammatory responses. The paradigm for EV-based therapy in AD may require a crucial deepening. This entails shifting the focus beyond modulating inflammatory phenotypes toward a new stage of immune cell reprogramming. This involves fundamentally reprogramming microglia from a harmful pro-inflammatory state to a protective state that efficiently phagocytoses Aβ plaques and actively promotes tissue repair.

### Gene editing

**Figure 3:**
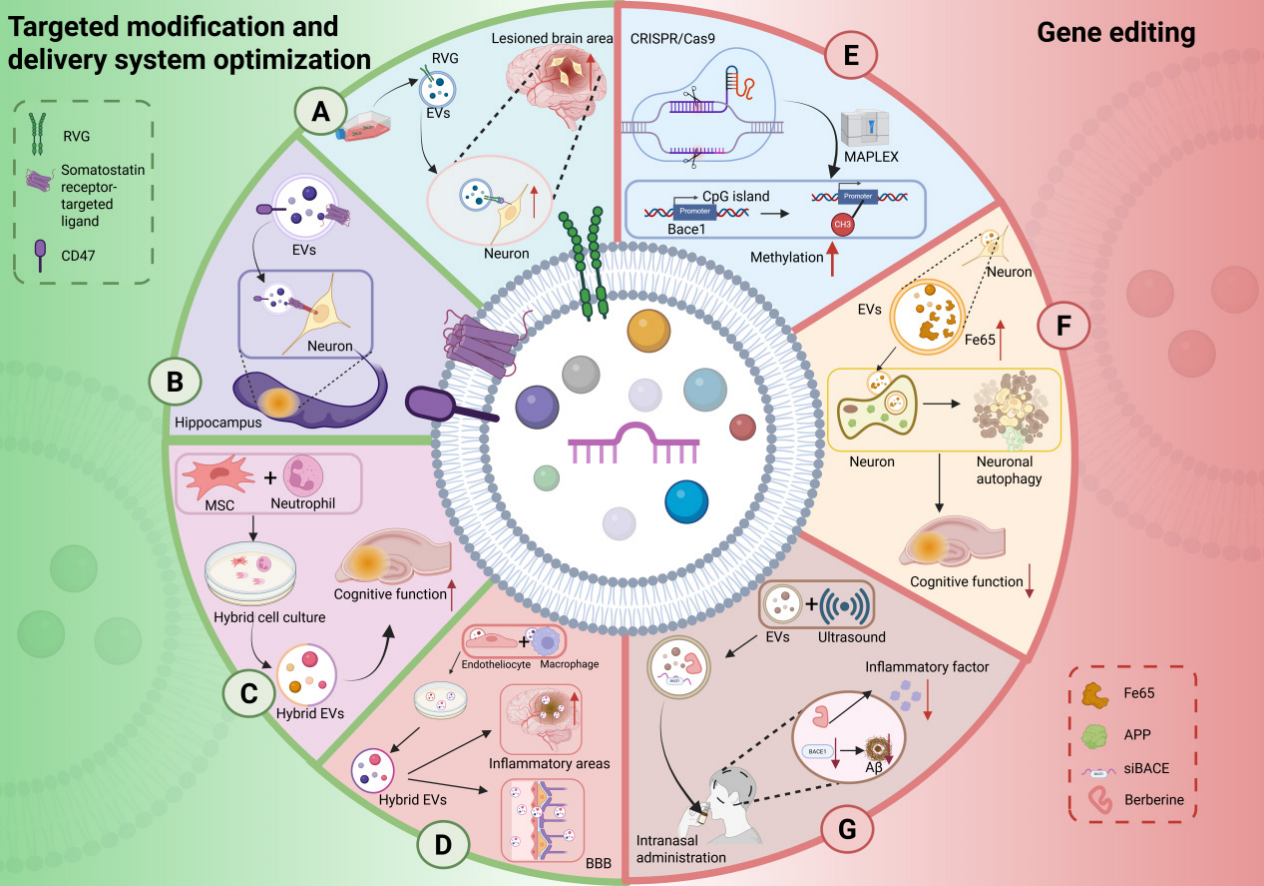
EV engineering strategies: targeted modification and gene editing. (A) Surface modification with RVG peptide enhances neuron-specific binding of EVs; (B) Expression of somatostatin receptor ligands enables hippocampus-specific EV delivery, while loading of CD47 ligands reduces their immune clearance and prolongs circulatory half-life; (C) EVs derived from mesenchymal stem cell-neutrophil hybrid cells target brain regions affected by AD; (D) Hybrid EVs generated by fusing membranes from brain microvascular endothelial cells and macrophage-derived EVs exhibit dual capabilities, which can efficiently cross the blood-brain barrier and accumulate in the inflamed areas of the brain, promoting the clearance of Aβ; (E) The CRISPR/Cas9 system is utilized to epigenetically target and methylate the BACE1 promoter, thereby reducing its expression; (F) Engineered hippocampal neuron-derived EVs overexpressing the Fe65 protein induce autophagy in APP-expressing neurons; (G) Ultrasound-mediated preparation generates EVs co-loaded with BACE1 siRNA and the anti-inflammatory drug berberine, enabling synergistic therapy upon intranasal administration. RVG: Rabies virus glycoprotein; EV: extracellular vesicle; MSC: mesenchymal stem cell; BBB: blood-brain barrier; MAPLEX: mMaple3-mediated protein loading into and release from exosomes; APP: amyloid precursor protein; Aβ: amyloid-beta; BACE1: beta-site amyloid precursor protein cleaving enzyme 1; CRISPR: clustered regularly interspaced short palindromic repeats; Cas9: CRISPR-associated protein 9; siRNA: small interfering RNA. Created in BioRender. yinghan, h. (2025) https://BioRender.com/br23jn3

In recent years, gene editing technology, particularly the clustered regularly interspaced short palindromic repeats (CRISPR)/CRISPR-associated protein 9 (Cas9) system, has provided powerful tools for engineering EVs. These tools can endow EVs with new biological activities and enable more precise and diverse functions. For example, the novel light-inducible protein delivery system, termed MAPLEX (mMaple3-mediated protein loading into and release from exosomes), was employed to target the promoter of the BACE1 and induce its methylation. This treatment significantly reduced beta-site amyloid precursor protein cleaving enzyme 1 (BACE1) expression levels in the brains of two distinct AD mouse models [five-times familial Alzheimer’s disease (5×FAD) and triple-transgenic Alzheimer’s disease (3×Tg-AD)]. This intervention subsequently improved recognition memory deficits and alleviated amyloid pathology^[190]^. Furthermore, engineered EVs can be modified through gene editing or functional engineering to deliver specific miRNAs, proteins, or other bioactive molecules. For instance, hippocampal neuron-derived EVs engineered to overexpress Fe65 induced autophagy in APP-expressing neuronal cells and ameliorated cognitive decline in AD mice^[22]^. In another study, researchers employed ultrasound-mediated methods to create EVs co-loaded with BACE1 siRNA and berberine. Following intranasal administration and delivery into the brain, these EVs reduced BACE1 expression and Aβ deposition, while the concurrently delivered berberine effectively suppressed the release of inflammatory factors, thereby mitigating neuroinflammation^[2]^. Optimizing the gene expression profiles and bioactivities of these multifunctional EVs through gene-editing technologies holds promise for further enhancing therapeutic efficacy and improving their adaptability for personalized treatment [Figure 3].

## EV PRE-TREATMENT

### Environmental pretreatment of EVs

In recent years, EVs have emerged as promising therapeutic vectors for AD treatment and neuroprotection, with their content regulation becoming a major research focus. Numerous studies have demonstrated that pre-treatment with hypoxia, drugs, or inflammatory factors can significantly alter the molecular profiles of miRNAs, circular RNAs (circRNAs), and proteins in EVs secreted by source cells, thereby enhancing their neuroprotective effects. Oxygen-deprived pretreatment of adipose-derived stem cells (ADSCs) enhances the enrichment of circular RNA enhancer of polycomb homolog 1 (circ-Epc1) in EVs. This process regulates the miR-770-3p/triggering receptor expressed on myeloid cells 2 (TREM2) axis, thereby promoting microglial transition from the pro-inflammatory M1 to the anti-inflammatory M2 phenotype, which alleviates cognitive impairment and neuronal damage in AD mouse models^[191]^.

Proteomic analyses further reveal that EVs from hypoxia-preconditioned BMSCs are enriched with autophagy-related proteins and promote the migration of normal BMSCs and tissue repair, offering important insights for neural regeneration studies^[91]^. Similarly, subjecting neural stem cells to a 42 °C heat shock alters the protein composition of their secreted EVs, enriching them with anti-apoptotic molecules and factors that promote DNA repair. These EVs exhibit enhanced antioxidant capacity and increased resistance to Aβ toxicity in *in vitro* AD models^[192]^.

Pharmacological pretreatment is a key strategy for regulating EV contents. For example, treating microglia with statins, such as lovastatin, promotes the secretion of insulin-degrading enzyme mediated by microglia-derived EVs, thereby enhancing Aβ degradation and reducing Aβ deposition^[193]^. Moreover, preconditioning MSC-EVs with traditional Chinese medicine (TCM) components, such as those from Salvia miltiorrhiza, upregulates hsa-miR-27a-5p_R-1 to modulate the signal transducer and activator of transcription 3 (STAT3)-SHANK2 (SH3 and multiple ankyrin repeat domains protein 2) axis, significantly improving the EVs’ anti-fibrotic and tissue-repair capabilities^[194]^.

**Figure 4:**
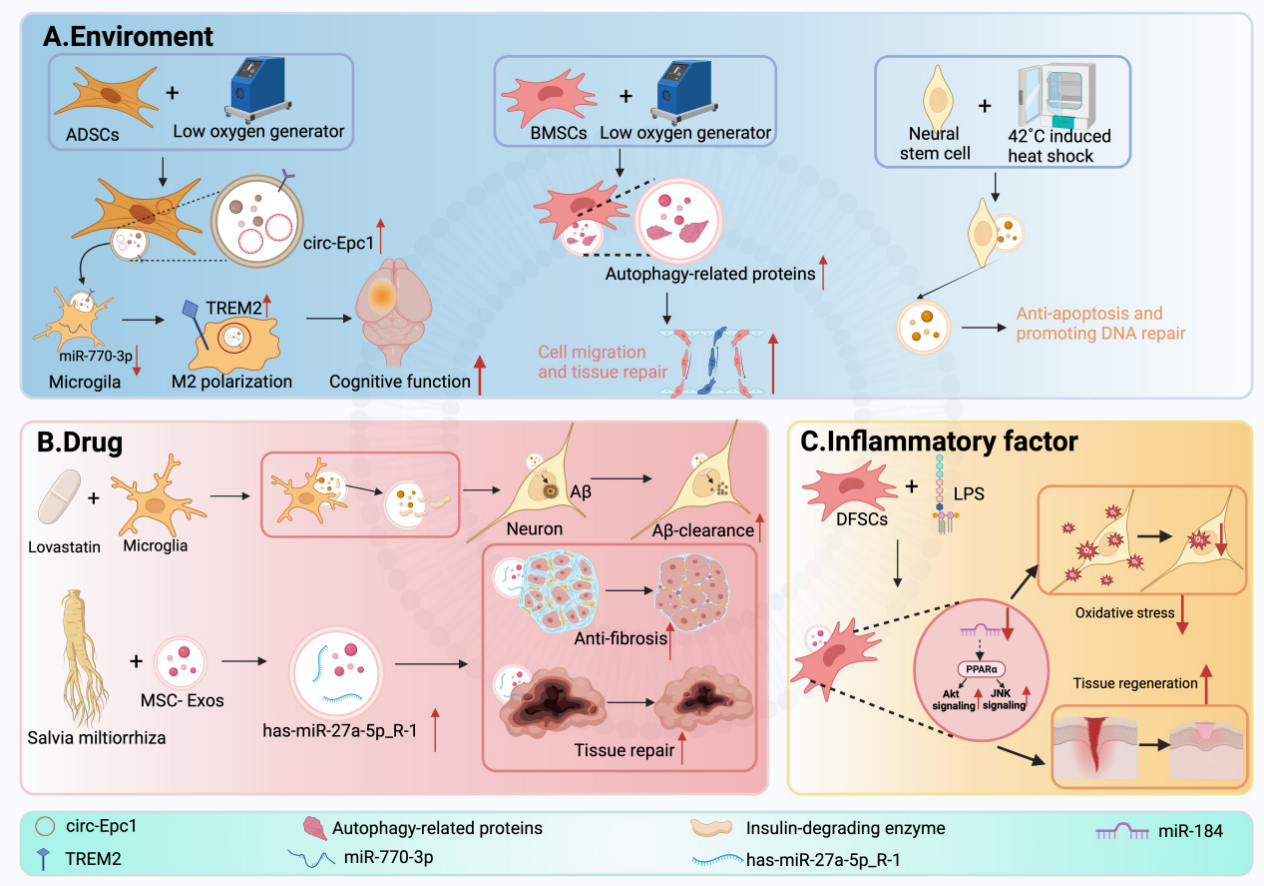
EV pre-treatment for enhanced therapeutic efficacy. (A) Environmental Preconditioning: Hypoxic preconditioning of ADSCs enriches circ-Epc1, promoting microglial polarization toward the anti-inflammatory M2 phenotype; hypoxic preconditioning of BMSCs enhances cell migration and tissue repair; heat shock treatment of neural stem cells increases anti-apoptotic and DNA repair capabilities, thereby augmenting the antioxidant capacity of the secreted EVs; (B) Pharmacological Preconditioning: Treatment of microglia with lovastatin promotes insulin-degrading enzyme secretion via their EVs; treatment of mesenchymal stem cells with Salvia miltiorrhiza extract upregulates miR-27a-5p, thereby enhancing Aβ degradation and tissue repair capabilities; (C) Inflammatory Factor Preconditioning: Stimulation of DFSCs with LPS downregulates miR-184 and activates the PPARα-Akt and JNK pathways, suppressing oxidative stress and promoting regeneration. ADSC: Adipose-derived stem cell; circ-Epc1: circular RNA enhancer of polycomb homolog 1; TREM2: triggering receptor expressed on myeloid cells-2; BMSC: bone marrow mesenchymal stem cell; Aβ: amyloid-beta; DFSC: dental follicle stem cell; LPS: lipopolysaccharide; PPARα: peroxisome proliferator-activated receptor alpha; EV: extracellular vesicle; JNK: c-Jun N-terminal kinase. Created in BioRender. yinghan, h. (2025) https://BioRender.com/p093jkc

Inflammatory cytokine preconditioning likewise influences EV composition. When dental follicle stem cells (DFSCs) are exposed to inflammatory factors such as lipopolysaccharide (LPS), the expression level of miR-184 in their EVs is downregulated, concurrently activating the PPARα (peroxisome proliferator-activated receptor alpha)-Akt (protein kinase B) and JNK (c-Jun N-terminal kinase) signaling pathway, suppressing oxidative stress, and promoting tissue regeneration^[195]^ [Figure 4].

### Optimization and challenges of preprocessing strategies

EV preconditioning strategies demonstrate considerable potential in treating AD and other neurodegenerative disorders, yet their optimization faces multiple challenges. When refining preconditioning protocols, ensuring functional consistency and controllability of EVs must be prioritized. Since the bioactivity of EVs is highly dependent on the state of the cell source and external stimuli, batch-to-batch variability remains a major obstacle to clinical translation. Therefore, it is necessary to standardize the pre-processing procedures, reduce the heterogeneity between batches, and establish a high-throughput quality control system. Some studies have already begun to identify specific EV components (such as specific miRNAs and proteins) as potential quality control biomarkers, providing a reference for clinical applications^[196]^.

Furthermore, it is worth noting that EV preconditioning not only influences biological function but may also exert dual effects on immunogenicity and *in vivo* biodistribution. While certain preconditioning methods can enhance EVs targeting and regenerative capacity, they may also trigger non-specific immune responses or accelerate systemic clearance, thereby affecting both therapeutic outcomes and safety profiles^[197]^. Therefore, future optimization of preconditioning strategies should comprehensively consider the immunomodulatory properties, *in vivo* stability, and long-term safety of EVs. This requires integrated evaluation using animal models and *in vitro* systems to gradually establish a controllable and traceable system for EVs preparation and application.

In summary, the optimization of EV pretreatment requires a systematic balance across multiple dimensions: cell source selection, processing conditions, functional consistency, and safety assurance. Through continued mechanistic investigation and the establishment of standardized operating procedures, a solid foundation will be laid for the clinical translation of EV-based therapies in AD and other neurodegenerative conditions.

## CONCLUSION

Based on the comprehensive review presented, EV-based therapeutics demonstrate transformative potential within AD research. While compelling efficacy and a favorable safety profile have been observed in preclinical studies and early-phase clinical trials, significant challenges remain. These include the standardization of isolation and purification protocols, the establishment of accurate dosing regimens, and the generation of robust long-term safety data. These hurdles underscore the complexity of translating EV-based therapies into clinical practice. Sustained investment in multi-omics research, engineering strategies, and translational science is imperative to translate this promising therapeutic frontier from the laboratory to the clinic, ultimately aiming to improve outcomes for patients afflicted by this devastating neurodegenerative disease.

## DECLARATIONS

### Copyright

© The Author(s) 2026.
